# Internet-Based Cognitive Behavioral Therapy in Stepped Care for Chronic Fatigue Syndrome: Randomized Noninferiority Trial

**DOI:** 10.2196/11276

**Published:** 2019-03-14

**Authors:** Margreet Worm-Smeitink, Anthonie Janse, Arno van Dam, Andrea Evers, Rosalie van der Vaart, Michel Wensing, Hans Knoop

**Affiliations:** 1 Department of Medical Psychology Radboud University Medical Center Nijmegen Netherlands; 2 Specialist Center for Complex Medically Unexplained Symptoms and Somatic Symptom Disorders Dimence Deventer Netherlands; 3 Department of Medical Psychology Amsterdam University Medical Centers University of Amsterdam Amsterdam Netherlands; 4 School of Social and Behavioural Sciences Tranzo Tilburg University Tilburg Netherlands; 5 Health, Medical and Neuropsychology Unit Institute of Psychology Leiden University Leiden Netherlands; 6 Department of Psychiatry Leiden University Medical Center Leiden Netherlands; 7 Department of General Practice and Health Services Research Heidelberg University Hospital Heidelberg Germany; 8 Radboud Institute of Health Sciences Radboud University Medical Center Nijmegen Netherlands; 9 Expert Center for Chronic Fatigue Department of Medical Psychology Amsterdam University Medical Centers, Vrije Universiteit Amsterdam Netherlands

**Keywords:** eHealth, chronic fatigue syndrome, cognitive behavioral therapy, randomized controlled trial

## Abstract

**Background:**

Internet-based cognitive behavioral therapy (I-CBT) leads to a reduction of fatigue severity and disability in adults with chronic fatigue syndrome (CFS). However, not all patients profit and it remains unclear how I-CBT is best embedded in the care of CFS patients.

**Objective:**

This study aimed to compare the efficacy of stepped care, using therapist-assisted I-CBT, followed by face-to-face (f2f) cognitive behavioral therapy (CBT) when needed, with f2f CBT (treatment as usual [TAU]) on fatigue severity. The secondary aim was to investigate treatment efficiency.

**Methods:**

A total of 363 CFS patients were randomized to 1 of the 3 treatment arms (n=121). There were 2 stepped care conditions that differed in the therapists’ feedback during I-CBT: *prescheduled* or *on-demand*. When still severely fatigued or disabled after I-CBT, the patients were offered f2f CBT. Noninferiority of both stepped care conditions to TAU was tested using analysis of covariance. The primary outcome was fatigue severity (Checklist Individual Strength). Disabilities (Sickness Impact Profile -8), physical functioning (Medical Outcomes Survey Short Form-36), psychological distress (Symptom Checklist-90), and proportion of patients with clinically significant improvement in fatigue were the secondary outcomes. The amount of invested therapist time was compared between stepped care and TAU. Exploratory comparisons were made between the stepped care conditions of invested therapist time and proportion of patients who continued with f2f CBT.

**Results:**

Noninferiority was indicated, as the upper boundary of the one-sided 98.75% CI of the difference in the change in fatigue severity between both forms of stepped care and TAU were below the noninferiority margin of 5.2 (4.25 and 3.81, respectively). The between-group differences on the secondary outcomes were also not significant (*P=*.11 to *P=*.79). Both stepped care formats required less therapist time than TAU (median 8 hours, 9 minutes and 7 hours, 25 minutes in stepped care vs 12 hours in TAU; *P*<.001). The difference in therapist time between both stepped care formats was not significant. Approximately half of the patients meeting step-up criteria for f2f CBT after I-CBT did not continue.

**Conclusions:**

Stepped care, including I-CBT followed by f2f CBT when indicated, is noninferior to TAU of f2f CBT and requires less therapist time. I-CBT for CFS can be used as a first step in stepped care.

**Trial Registration:**

Nederlands Trial Register NTR4809; http://www.trialregister.nl/trialreg/admin/rctview.asp?TC=4809 (Archived by WebCite at http://www.webcitation.org/74SWkw1V5)

## Introduction

Chronic fatigue syndrome (CFS), sometimes referred to as myalgic encephalomyelitis (ME), is characterized by medically unexplained, severe, ongoing, and disabling fatigue. It is not alleviated by rest, and according to the revised US Centers for Disease Control (CDC) criteria from 2003, it is accompanied by at least 4 out of the following 8 additional symptoms: postexertional malaise, headache, unrefreshing sleep, muscle pain, joint pain, sore throat, tender lymph nodes, and impaired concentration or memory [1,2]. The prevalence of CFS is estimated to be around 1% [3] and the prognosis is unfavorable without treatment; the median spontaneous recovery rate is 5% [4]. It is unknown what causes CFS, and it is commonly assumed to be multifactorially determined.

It is helpful to distinguish between precipitating and perpetuating factors of CFS [5]. Precipitating factors might be a virus infection or a stressful life event that triggers severe fatigue. Perpetuating factors are thought to maintain the fatigue, even when the precipitating factor is no longer present. According to the cognitive behavioral model of CFS, these perpetuating factors are behavior and beliefs [6,7]. Cognitive behavioral therapy (CBT) aims to change these beliefs and behaviors and is found to lead to a significant reduction of fatigue and disability [8-10].

CBT for CFS is a time-intensive treatment, requiring 12 hours of therapist contact on average [10]. This is a problem, as treatment capacity is limited [11]. A possible solution is using internet-based CBT (I-CBT). I-CBT often takes less therapist time to deliver, increasing the number of patients that can be treated [12-14]. I-CBT has a number of other advantages over face-to-face (f2f) CBT. It reduces the traveling time and the need to schedule appointments, which relieves some of the burden of treatment for patients [13]. The treatment is accessible whenever patients want [12,13], which may be empowering [14]. Furthermore, I-CBT might increase motivation, as it offers a wide range of attractive audiovisual information elements and the possibility to receive feedback on the progress made [13].

In the past years, I-CBT was developed for several mental disorders and chronic medical conditions, and its efficacy has been tested in randomized controlled trials (RCTs) [12-14]. Systematic reviews seem to indicate that guided I-CBT can reach effect sizes equivalent to those found in f2f CBT [15,16]. However, I-CBT was not often directly compared with a more traditional, high-intensity f2f CBT. Additionally, I-CBT is not yet available for all medical and psychiatric conditions.

We developed I-CBT for adults with CFS and recently tested its efficacy in an RCT. I-CBT led to a reduction of fatigue and disabilities compared with a waiting list [17]. Approximately 40% of the patients had a clinically significant and reliable change in fatigue severity and were no longer severely fatigued after I-CBT. To gain more insight into the role of therapist feedback in I-CBT, 2 delivery formats of I-CBT were tested, which differed in therapist guidance. In the *protocol-driven feedback* format, the therapists’ feedback was scheduled with preset time intervals. The therapist sent reminders if the schedule was not adhered to. In the *feedback on demand* format, feedback was only given when the patient asked for it. Both the I-CBT formats were equally effective, but the feedback-on-demand version required significantly less therapist time [17].

It is important to uncover how I-CBT can best be embedded in clinical care for CFS. The effect size of I-CBT on fatigue severity was smaller than the effect size previously found in a study investigating the efficacy of f2f CBT delivered in groups, using the same treatment principles (effect sizes 0.6 and 1.1, respectively) [17,18]. This suggests that I-CBT is less effective than f2f CBT. A straightforward solution would be to blend I-CBT with f2f CBT. Unfortunately, there is a lack of data to conclude that combinations of internet-based and f2f CBT are as effective as established f2f treatments. In addition, it is not known what an optimal format of blending of both treatment formats would be (eg, nonsequential vs sequential) [19]. We chose to embed I-CBT in a stepped care approach, as a first step. Patients who did not profit from I-CBT, as indicated by severe fatigue or disability still present following I-CBT, would step up to f2f CBT. If stepped care is as effective as treatment as usual (TAU; ie, f2f CBT), this would imply that I-CBT can be used in routine clinical care for CFS. Treatment intensity would then be decreased for the substantial group of patients for which I-CBT suffices. Furthermore, stepped care may be more time-efficient than TAU, in that less therapist time would be needed to deliver stepped care [20]. This could increase cost-effectiveness.

The primary objective of this study was to determine whether stepped care for CFS, with I-CBT as the first and f2f CBT as the second step, would be as effective as TAU. This randomized noninferiority trial was a follow-up study of the RCT investigating the efficacy of I-CBT for adults with CFS. All patients who were still severely fatigued or functionally impaired after I-CBT or the waiting list were offered f2f CBT, resulting in 2 stepped care conditions and 1 TAU arm. The efficacy in reduction of fatigue and efficiency in reduction of therapist time needed for stepped care were compared with TAU.

Moreover, we explored the role of therapist guidance in I-CBT, as it is not well understood how therapist feedback influences I-CBT. Guided I-CBT was found to be more effective than unguided I-CBT [21-23], but it is not known how much guidance is needed, who needs to provide the guidance [23,24], and what aspect of the guidance is (most) helpful [22]. In our previous study, we found that whether therapist feedback was *prescheduled* or *on-demand* did not influence the treatment effect [17]. We explored in this study how therapist guidance during I-CBT influenced the outcome of stepped care. More specifically, (1) whether the difference in therapist time needed between the I-CBT arms persisted in stepped care and (2) whether the proportion of patients stepping up to f2f CBT after I-CBT differed in both formats, as the format of therapist feedback may influence the willingness to step up to f2f CBT.

## Methods

### Trial Design

This study was registered in the Dutch trial register (NTR4809) and was approved by the medical ethical committee of the Radboud University Medical Center (reference NL42543.091.12). It was a follow-up study of an RCT testing the efficacy of I-CBT for CFS (NTR4013) and was designed as a three-arm, parallel, randomized, noninferiority trial. In 2 arms, the patients received stepped care (SC) consisting of I-CBT, either with protocol-driven feedback (SC-protocol-driven feedback) or with feedback on demand (SC-feedback-on-demand), followed by f2f CBT when necessary, that is, still severely fatigued (CIS fatigue severity >35) or disabled (SIP >700) after I-CBT. The third arm was f2f CBT after a variable waiting period (TAU).

Before randomization, all patients completed a baseline assessment (T0). Directly after randomization, the patients in the stepped care arms started with I-CBT for a duration of 6 months. After 6 months, they completed a second assessment (T1). If they started additional f2f CBT after T1, they were assessed again 6 months later (T2). Patients from the TAU group were placed on the waiting list directly after T0, for a duration of maximally 6 months (refer to the section Interventions), after which they completed their T1 assessment. Directly after T1, the f2f CBT was started. Furthermore, 6 months after T1, the patients were assessed again (T2). Assessments were web-based questionnaires.

#### Participants

Participants were all recruited from adult CFS patients consecutively referred to a tertiary CFS treatment center at a university hospital. They comprised the 240 participants of the RCT testing the efficacy of I-CBT [17] and 123 participants additionally randomized to gain sufficient power for the primary research question of this study. It was a closed study; only patients referred to the treatment center could participate. All patients were first seen by a consultant of the outpatient clinic of the department of Internal Medicine of the hospital. The consultant checked the medical status to confirm that patients were sufficiently examined and if not, they examined the patients according to the national CFS guidelines [25]. Patients meeting CDC criteria for CFS [1,2] underwent a clinical assessment at the treatment center, including a structured interview (the Mini-International Neuropsychiatric Interview [26]), to rule out psychiatric disorders that could explain the presence of fatigue. Comorbidities that could not explain fatigue were not exclusion criteria.

Eligible patients were informed about the study and were included after giving written informed consent. They were asked to refrain from seeking treatment for CFS elsewhere for the duration of the study. Inclusion criteria were being aged 18 years or above; ability to speak, read, and write Dutch; meeting the CDC criteria, revised in 2003, for CFS [1,2]; severely fatigued (checklist Individual strength subscale, fatigue severity score of ≥35 [27]); severely disabled (Sickness Impact Profile 8, total score of ≥700 [28]); able to use a computer and have access to the internet; and given written informed consent. Exclusion criteria were being involved in a legal procedure concerning disability/benefit claims and/or participating in other CFS research [29].

#### Interventions

##### Treatment as Usual (Direct Face-to-Face Cognitive Behavioral Therapy)

For patients in the TAU group, the therapy started after a waiting period. During the first part of the study (first 240 randomized patients), the waiting list was 6 months for all patients. In the second part of the study (last 123 patients), the waiting period fluctuated depending on treatment capacity but was not longer than 6 months. For ethical reasons, patients could start sooner if the waiting period for routine clinical care was less than 6 months.

CBT was delivered according to a treatment protocol [30] that was also used in previous RCTs [18,20]. The treatment aims at changing fatigue-related beliefs and behavior. CBT starts with educating the patients on the cognitive-behavioral model of CFS and formulating treatment goals which, when attained, imply recovery from CFS. Following this, the patient learns to adopt a regular sleep-wake cycle, with fixed bed times and without sleep during the day. The patient is taught to shift attention away from fatigue and to challenge unhelpful beliefs regarding fatigue and disability. This is followed by a graded activity program in which a systematic increase in physical activity, regardless of symptoms, usually by walking or cycling, is introduced. Patients are encouraged to challenge dysfunctional beliefs about symptoms and activity during the program. The graded activity program is tailored to the activity pattern of the patient: relatively active patients learn to spread activity evenly first, whereas less active patients start directly with graded activity. An actometer, a motion-sensing device, was used to determine the activity pattern [29,31]. After the increase in physical activity, the patients learn to use these principles to increase mental and social activity. After the graded activity program, the patient is taught to reach his personal goals step by step. Finally, the patient is encouraged to experiment with fluctuating bedtimes and activity levels and to adopt a healthy view on normal fatigue.

##### Stepped Care

The I-CBT was accessible via a website, with a username and password that the patient received by email. Patients did not have to pay to use the intervention, although internet access was not provided. Patients were not trained to use the platform and could ask for support by email. During the f2f diagnostic sessions, the patient had met the therapist who would deliver the I-CBT. Both versions of I-CBT were based on the treatment protocol for f2f CBT for CFS [17,30]. The conditions differed in when the patient received feedback. In SC protocol-driven feedback, during I-CBT, the therapist asked the patient to report on the progress made, on fixed time points: weekly in the first 4 weeks and fortnightly in the following 8 weeks. From week 13, the frequency could be lowered to once every 3 weeks, if enough progress was being made. The therapist gave feedback via email and sent a reminder if no update was received. The feedback was aimed at motivating the patient to put the instructions of the intervention into practice. In the *feedback-on-demand* format, patients received feedback only when they indicated a need for advice. No reminders were sent.

The I-CBT consisted of 7 modules: (1) In “getting started and goal setting” psychoeducation is given, a treatment contract is signed, and goals are set. When goals are submitted, the next 5 modules are accessible: (2) “regulate sleep-wake cycle,” (3) “helpful beliefs about fatigue,” (4) “how to communicate with others about CFS,” (5) “gradually increasing my activity,” and (6) “reaching my goals step by step.” When this module is completed, the last module becomes accessible: (7) “evaluation and the future.” The modules are described in more detail elsewhere [29].

Within 2 weeks after T1, patients had an f2f evaluation session with their therapist. During this session, it was evaluated whether treatment goals were reached or additional f2f CBT was indicated. Patients were offered f2f CBT after I-CBT if they were still severely fatigued (CIS fatigue severity ≥35) and/or severely disabled (SIP8 ≥700) and/or if not all therapy goals were attained and the therapist expected that additional treatment was necessary to attain them. This decision could only be made after consultation of an experienced supervising CBT therapist (HK). The reason for continuation was, in that case, recorded and reported.

The additional CBT was delivered according to the treatment protocol [30]. It evaluated which beliefs and behaviors were already changed and consequently, which interventions were still needed to make further progress. Therapists were trained to encourage patients to step up when still fatigued or disabled after I-CBT. They were instructed to avoid that patients conclude that the I-CBT failed and additional f2f CBT will not lead to a (further) reduction of symptoms. The therapists helped patients appreciate what was already achieved and clarify that the f2f CBT is tailored to what is needed to make further gains.

Therapists in all the 3 arms were psychologists, trained in CBT for CFS, both f2f and internet-based. Therapists received weekly group supervision during the study [29].

### Measures

#### Baseline Characteristics

The following patient characteristics were recorded: age, sex, duration of symptoms, the presence of each CDC symptom, work status, years of education followed, and the presence of depressive symptoms above a clinical significant cutoff of 4 on the Beck Depression Inventory, primary care version [32]. Furthermore, it was recorded whether patients met the SEID (systemic exertion intolerance disease) criteria [33]. SEID was recently proposed by the U.S. Institute of Medicine as an alternative to ME/CFS. SEID was met when postexertional malaise, unrefreshing sleep, and memory/concentration problems were all reported [33]. SEID can also be confirmed when a patient has orthostatic intolerance instead of memory/concentration problems, aside from severe and persistent fatigue. However, orthostatic intolerance was not assessed.

#### Primary Outcome Measure: Fatigue Severity

Fatigue severity (the primary, noninferiority outcome) was measured with the Checklist Individual Strength (CIS) fatigue severity subscale [27,34]. The CIS contains 20 items, Likert scaled (from 1-7) assessing 4 aspects of fatigue. The fatigue severity subscale is often used as a measure of fatigue in studies on CBT for CFS and is reliable and valid [27]. It contains 8 items, scores range from 8 (not fatigued) to 56 (severely fatigued). A validated cutoff of 35 was used to indicate severe fatigue [35].

#### Secondary Outcome Measures

##### Disabilities

A total score of Sickness Impact Profile 8 (SIP8; [28]) measures disabilities at 8 domains of daily functioning. Patients can indicate which out of 86 statements apply to their functioning as a result of their health status. Each statement has a weighting factor indicating severity [28]. The weighed total score was used. Higher scores indicated more severe disabilities. In this study, a total score of ≥700 was used as a cutoff to indicate significant disability.

##### Physical Functioning

The Medical Outcome Survey Short Form-36 (SF-36; [36,37]) physical functioning subscale was used to measure self-reported physical functioning and ranged from 0 (maximum limitations) to 100 (no limitations).

##### Psychological Distress

The Symptom Checklist-90 (SCL-90 [38]) total scale score measures psychological distress with 90 items, answered on a 5-point Likert scale (range of 90-450). Higher scores indicate more distress.

##### Invested Therapist Time

Therapists recorded the time needed per patient. Time spent on I-CBT (in stepped care), on additional telephone calls or email contact (all conditions) were recorded in minutes. Each f2f CBT session counted for 60 min (all conditions). In all conditions, 120 min was counted for the diagnostic assessment, consisting of 2 sessions.

### Sample Size

Assuming a power of 0.80, a one-sided alpha of .0125 (correcting for 2 comparisons), an SD of 13.6 [20], and a noninferiority limit of 5.2 units on the CIS, 108 patients were needed per arm. To account for an expected dropout rate of 10.7%, the sample size needed for each arm was 121, making the total number of patients needed to 363. Therefore, 123 patients were to be randomized in addition to the 240 who were already randomized. The dropout rate of 10.7% was found in a study investigating stepped care for CFS with a self-help booklet as the first step [20]. A one-sided interval was used, as we tested for noninferiority. The 5.2 noninferiority margin is the estimated average decrease on the CIS fatigue severity subscale that occurs during waiting list, which is assumed to be clinically nonsignificant [39].

### Randomization and Blinding

Patients were randomly allocated to 1 of the 3 conditions. Randomization was computer-generated, in blocks of 12 patients. The randomization program was programmed by a statistician, not involved in this study. Randomization was performed by an administrative assistant, in the presence of the patient and the therapist. Participants were partly blinded: they were unaware of the existence of 2 I-CBT formats and were told that they could either receive I-CBT followed by CBT if needed or f2f CBT. After randomization, therapists and patients could read the result from the computer screen: “(1) internet therapy” and “(2) internet therapy” or “wait list.” The therapist knew that condition “1” of I-CBT was protocol-driven feedback and “2” was feedback on demand.

Statistical analysis was performed on a data file, which blinded the researcher performing the analysis to patient and allocation condition. Post analysis, allocation to condition was unmasked to enable the authors to interpret the results.

### Statistical Analyses

Analyses were done on the basis of intention to treat after imputation of missing primary and secondary outcome measures at postassessment. Postassessment was T1 for patients who stopped after waiting list or after I-CBT and was T2 for patients who received (additional) f2f CBT. When T2 data were needed but only T1 was present, postassessment scores were imputed as well. We used multiple imputation (20 imputed data sets), assuming that data were missing at random. All outcome variables at postassessment were included in the imputation model and were imputed. Baseline variables of all outcomes were only entered as predictors for the imputation model and were not imputed. Imputation was done in IBM SPSS version 22, as were all statistical analyses.

#### Outcomes of Both Versions of Stepped Care Compared With Direct Face-to-Face Cognitive Behavioral Therapy

To answer the primary research question, an analysis of covariance (ANCOVA) was performed with postassessment CIS fatigue severity score as the dependent variable, baseline fatigue scores as covariate, and treatment condition as the fixed factor. Noninferiority was assumed when post-treatment CIS fatigue severity was maximally 5.2 points higher for either form of stepped care in comparison with TAU [20]. Hence, the upper bound of the one-sided 98.75% CI of the difference between the formats should be no larger than 5.2. To compare the effect on disabilities, physical functioning, and psychological distress of stepped care and TAU, ANCOVAs were performed for SF-36, SIP8, and SCL-90, with baseline of the dependent variable as covariate and condition as fixed factor.

Differences in the proportion of patients with clinically significant improvement in fatigue severity were compared with chi-Square tests. Each stepped care group was compared with TAU. Clinically significant improvement in fatigue severity was assumed when there was a statistically reliable change of >1.96 SD in combination with a CIS fatigue scores of <35 on postassessment. The reliable change index (RCI) of the CIS fatigue severity was calculated for each person following Jacobson and Truax [40]. For each condition, the SD used in the formula was the SD of the baseline CIS fatigue severity score in the specific condition. The reliability of the CIS used in the calculation was 0.88 [34]. An RCI larger than 1.96 indicates that with a CI of 95%, it can be assumed that the improvement in CIS fatigue severity represents a true change and is not the consequence of the unreliability of the measure.

In addition, 3 sensitivity analyses were performed. First, the noninferiority was tested, whereas missing observations on fatigue severity were not imputed with multiple imputations but replaced in the following manner: (1) it was hypothesized that patients in the TAU group improved, missing scores were replaced by the CIS fatigue severity score at T0 minus the mean change in fatigue of the TAU group and (2) it was hypothesized that patients of the stepped care groups deteriorated, scores were replaced by the maximum CIS fatigue severity score. Second, the main analysis was repeated excluding patients who received CBT by telephone or email, instead of f2f. Third, the main analysis was repeated selecting only patients who met the SEID criteria.

#### Therapist Time Invested

Therapist time of both stepped care conditions were compared with TAU (either means with *t* tests or medians with Mann-Whitney *U* tests, when not normally distributed). The analysis was done with 2 selections: (1) including all patients: patients who did not start therapy only accounted for 120 min for the diagnostic assessment sessions, (2) including only “starters”: for the stepped care conditions, this was defined as having logged in the I-CBT program for at least three times and having submitted treatment goals [29] or having started f2f CBT. For TAU, it was defined as having received at least one therapy session. For patients who were still in treatment at the end of the study, the therapist time and therapy duration until that point were used. This was at least 1 year after randomization and at least 6 months after the start of f2f CBT. In addition, therapist time spent in both stepped care formats was compared.

#### Additional Effect Face-to-Face Cognitive Behavioral Therapy After Internet-Based Cognitive Behavioral Therapy

For patients in the stepped care groups who received additional f2f CBT, whether treatment outcomes at T2 (after f2f CBT) differed significantly from T1 (after I-CBT) was investigated using paired-samples *t* tests.

#### Outcome Differences Between First 240 and Last 123 Randomized Patients

This analysis compared 2 groups of patients. The first 240 randomized patients were included between April 2013 and June 2015 [17] and the second 123 subsequently, between June 2015 and December 2016. Both took place in different circumstances; during the second part of the study, the treatment center was unexpectedly moved to another university medical center, which resulted in therapist changes and increased travel time for patients. Treatment outcome for all measures were compared with *t* tests.

## Results

### Overview

**Figure 1:**
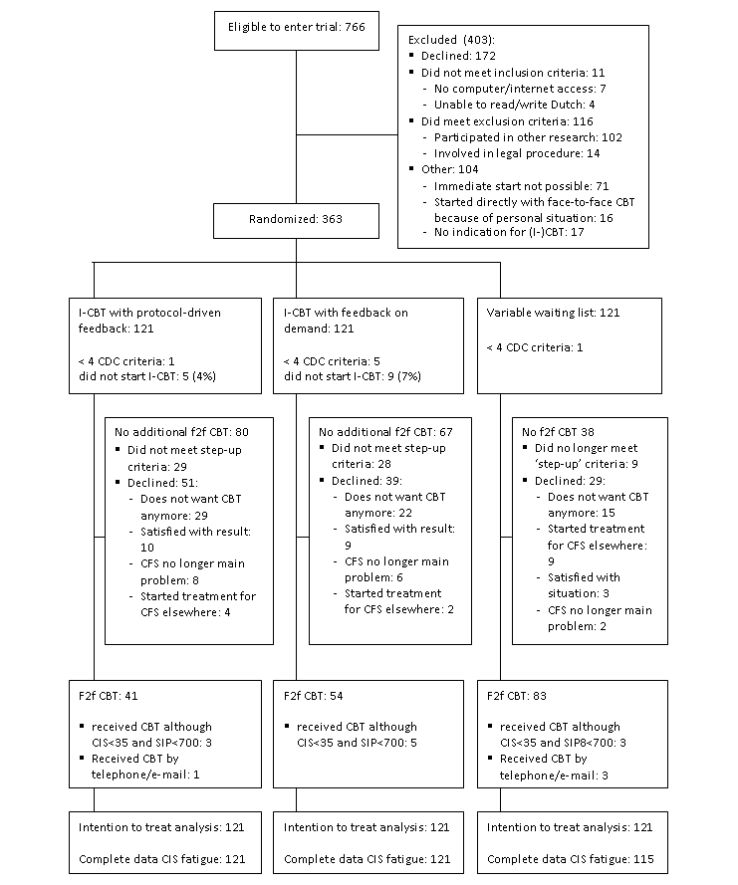
CONSORT flow diagram of eligibility criteria. CBT: cognitive behavioral therapy; CDC: Centers for Disease Control and Prevention; CFS: chronic fatigue syndrome; f2f: face-to-face; I-CBT: internet-based cognitive behavioral therapy.

Patients were recruited between April 2013 and December 2016 and data collection was completed in December 2017. As shown in the flowchart (Figure 1), out of 766 patients screened for eligibility, 363 patients were randomly assigned to 3 treatment arms. Of these, 7 randomized patients did not meet the inclusion criteria, as the number of additional CDC criteria was less than 4.

In the TAU condition, the waiting list duration was 29 weeks (SD 4) for the first part of the study and 18 weeks (SD 12) for the second part of the study. The Spearman rank correlation between waiting list duration and reduction of fatigue was *r*_s_=0.075, *P*=.50. After waiting list, 5 patients were lost to follow-up. Moreover, 12 patients did no longer meet the step-up criteria (severely fatigued as indicated by CIS fatigue severity >35 and/or severely disabled indicated by SIP8>700). In addition, 3 of them started individual CBT. Out of the remaining 104 patients, 77 started f2f CBT and 3 received CBT via telephone/email.

In the SC-protocol-driven feedback condition, 116 out of the 121 patients started I-CBT (95.8%). At T1, 87 patients met the step-up criteria (71.9%). Of them, 37 (43%) started f2f CBT. Furthermore, 3 patients, who did not meet the step-up criteria, received f2f CBT because several goals were not reached. In addition, 1 patient received the additional CBT by telephone and email.

In the SC feedback-on-demand condition, 113 patients started I-CBT (93.3%). Of the 85 patients who met the step-up criteria (70.2%), 48 (56%) started f2f CBT. Moreover, 6 patients who did not meet the step-up criteria received f2f CBT because not all treatment goals were attained.

The difference in the proportion of patients meeting the step-up criteria that did actually step up to f2f CBT between both stepped care conditions was not significant (χ^2^_1_=3.3; *P*=.07). Reasons for not stepping up were registered (see Figure 1): in the SC-protocol-driven feedback format, 29 (57%) did not want CBT anymore (15 because of the nature of the intervention, eg, did not want to travel, 14 could not fit f2f CBT in their lives at that moment eg, because of pregnancy), 10 (20%) were satisfied with the result of I-CBT, 8 (16%) no longer viewed CFS as the main problem, and 4 (8%) started treatment elsewhere. In SC-feedback-on-demand, 22 patients (56%) did not want CBT (17 because of the nature of the intervention and for 5 it did not fit into their lives), 9 (23%) were satisfied with the result of I-CBT, 6 (15%) no longer viewed CFS as the main problem, and 2 (5%) started treatment elsewhere.

At the end of the study, 1 patient was still in therapy and completed the postassessment 6 months after the start of f2f CBT. None of the patients were excluded from the main analysis. Table 1 shows the baseline characteristics.

### Outcomes of Stepped Care Compared With Treatment As Usual

#### Noninferiority With Respect to Effect on Fatigue Severity

Data on the primary outcome were missing for 6 patients (all in the TAU condition). The upper boundary of the one-sided 98.75% CI of both forms of stepped care was below 5.2, indicating noninferiority. The mean difference in fatigue severity for the SC-protocol-driven feedback condition with TAU was −0.04 points; the CI upper bound was 3.81. For the SC-feedback-on-demand condition, the difference was 0.41 points; the upper bound of the CI was 4.25.

In all sensitivity analyses, both stepped care conditions remained noninferior to TAU (upper boundary CI for SC-protocol-driven feedback ranging from 3.34-3.95; upper bound CI SC-feedback-on-demand ranging from 4.12-4.50).

#### Secondary Outcome Measures

There were no significant differences between either stepped care condition or TAU for all secondary outcomes, that is, level of disabilities (SIP8), physical functioning (SF-36 physical functioning), and psychological distress (SCL-90). Within-group effect sizes are provided in Table 2.

#### Proportion of Patients With Clinically Significant Improvement in Fatigue Severity

Post stepped care, 49 out of 121 (40%) patients in SC-Protocol-driven feedback during I-CBT showed clinically significant improvement in fatigue severity. In the TAU group, 53 out of 115 patients improved. Assuming that patients with missing data did not improve, this was 44%. The difference between both groups was not significant; χ^2^_1_(N=236)=0.7; *P*=.39.

In SC-feedback-on-demand, 61 patients improved (50%). The difference between SC-feedback-on-demand and TAU was also not significant: χ^2^_1_(N=236)=0.4; *P*=.51.

### Therapist Time Invested

As the data on therapist time were not normally distributed, medians were compared with Mann-Whitney *U* tests (see Table 3). The intention-to-treat analysis showed that the median therapist time invested did not differ significantly between SC-protocol-driven feedback and TAU (*U*=7068.0; *P*=.64) and for SC-feedback-on-demand and TAU (*U*=7272.5; *P*=.93).

The analysis including only patients who started treatment showed different results. Median therapist time of both the stepped care formats differed significantly from TAU (*U*=6819.5; *P*<.001 for SC-protocol-driven feedback and *U*=6883.5; *P*<.001 for SC-feedback-on-demand).

**Table 1:** Baseline characteristics.

Baseline characteristic	Stepped care		Treatment as usual
	Protocol-driven feedback	Feedback on demand	
Age in years, mean (SD)	36.6 (12.8)	37.2 (12.3)	38.7 (12.5)
Female (N=121), n (%)	78 (64.5)	69 (57.0)	74 (61.2)
Paid job (N=119), n (%)	82 (68.9)	79 (65.8)	77 (64.7)
Education level in years, mean (SD)	15.4 (1.9)	14.8 (2.3)	15.7 (1.5)
Duration of complaints in years, median (IQR^a^)	4.0 (8.0)	5.0 (12.0)	6.0 (9.0)
Number of CDC^b^ symptoms, median (IQR)^c^	6 (2)	6 (3)	6 (2)
Memory and/or concentration problems (N=121), n (%)	114 (94.2)	112 (92.6)	116 (95.9)
Sore throat (N=121), n (%)	53 (43.8)	57 (47.1)	56 (46.3)
Tender lymph nodes (N=121), n (%)	49 (40.5)	61 (50.4)	52 (43.0)
Muscle pain (N=121), n (%)	91 (75.2)	97 (80.2)	99 (81.8)
Multi-joint pain (N=121), n (%)	83 (68.6)	86 (71.7)	93 (76.9)
Headaches (N=121), n (%)	91 (75.2)	94 (77.7)	85 (70.2)
Unrefreshing sleep (N=121), n (%)	119 (98.3)	114 (94.2)	119 (98.3)
Postexertional malaise (N=121), n (%)	113 (93.4)	109 (90.1)	113 (93.4)
Meeting SEID^d^ criteria (N=121), n (%)	89 (73.6)	92 (76.0)	93 (76.9)
Fatigue severity^e^, mean (SD)	50.8 (5.0)	50.2 (4.8)	49.7 (5.3)
Overall impairment^f^, mean (SD)	1488.6 (550.1)	1534.7 (562.0)	1599.2 (589.6)
Physical functioning^g^, mean (SD)	62.3 (20.1)	60.5 (19.4)	61.0 (20.4)
Psychological distress^h^, mean (SD)	154.4 (31.8)	160.2 (37.7)	161.2 (38.0)
Clinically relevant depressive symptoms^i^ (N=120), n (%)	42 (35.0)	39 (32.5)	44 (37.0)^j^
No current psychiatric diagnosis^k^ (N=121), n (%)	100 (83)	103 (85)	99 (82)
Any depressive disorder^k^ (N=121), n (%)	11 (9)	9 (7)	14 (12)
Any anxiety disorder^k^ (N=121), n (%)	11 (9)	11 (9)	8 (7)
Other psychiatric disorder^k^ (N=121), n (%)	1 (1)	1 (1)	4 (3)
Pain^g^, mean (SD)	59.3 (25.5)	59.1 (25.5)	57.7 (25.2)

^a^IQR: interquartile range.

^b^CDC: Centers for Disease Control and Prevention.

^c^Memory and/or concentration problems were scored together, so a maximum of 8 symptoms was scored.

^d^SEID: systemic exertion intolerance disease.

^e^CIS: Checklist Individual Strength.

^f^SIP8: Sickness Impact Profile.

^g^SF-36: Medical Outcomes Survey Short Form-36.

^h^SCL-90: Symptom Checklist-90.

^i^BDI-PC: Beck Depression Inventory-PC; total score ≥4.

^j^N=119.

^k^MINI: The Mini-International Neuropsychiatric Interview.

**Table 2:** Treatment effects.

Outcome measure		Stepped care		Treatment as usual
		Protocol-driven feedback	Feedback-on-demand	
**CIS^a^** **fatigue severity**				
	Pre	50.78	50.20	49.69
	Post	35.60	35.68	34.94
	Cohen *d* (95% CI)^b^	1.44 (1.16-1.73)	1.50 (1.21-1.79)	1.41 (1.12-1.69)
**SIP8^c^**				
	Pre	1488.56	1534.74	1593.20
	Post	822.09	797.10	961.32
	Cohen *d* (95% CI)^b^	1.09 (0.82-1.36)	1.22 (0.94-1.50)	0.91 (0.65-1.18)
**SF-36^d^** **physical functioning**				
	Pre	62.27	60.54	60.95
	Post	75.34	77.82	76.54
	Cohen *d* (95% CI)^b^	0.58 (0.33-0.84)	0.86 (0.59-1.12)	0.72 (0.46-0.98)
**SCL-90^e^**				
	Pre	154.36	160.20	161.22
	Post	137.69	140.79	143.65
	Cohen *d* (95% CI)^b^	0.42 (0.17-0.68)	0.46 (0.21-0.72)	0.40 (0.14-0.65)

^a^CIS: Checklist Individual Strength.

^b^Uncontrolled effect size: within-group effect. Cohen *d*=(Mean_pre_−Mean_post_/pooled SD).

^c^SIP8: Sickness Impact Profile 8.

^d^SF-36: Medical Outcomes Survey Short Form-36.

^e^SCL-90: Symptom Checklist-90.

**Table 3:** Therapist time invested in total treatment in hours.

Treatment arm	Intention to treat				Starters only			
	n	Mean (hours)	Median (hours)	Minimum-maximum (hours)	n	Mean (hours)	Median (hours)	Minimum-maximum (hours)
Stepped care–protocol-driven feedback	121	09:10	08:00	2:55-22:20	118	09:19	08:09	2:55-22:20
Stepped care–feedback-on-demand	121	08:30	06:55	2:00-21:45	117	08:42	07:25	2:00-21:45
Treatment as usual	121	08:54	09:00	2:00-27:00	83	12:03	12:00	4:00-27:00

### Exploratory Comparison of Both Stepped Care Formats

The difference in time invested between both the stepped care versions was significant when analyzing data of all patients (*U*=6237.0; *P*=.047), but the difference failed to reach significance when selecting only starters (*U*=5918.0; *P*=.06). More detailed information on the therapist time is provided in Table 4. On average, in SC-protocol-driven feedback, 4:04 hours (SD 2:20 hours) were spent on I-CBT. Moreover, 40 patients received f2f CBT, which took an average 6:18 hours (SD 3:37 hours) per person who received it. In SC-feedback-on-demand, 2:29 hours (SD 2:28 hours) was spent on I-CBT. In addition, 54 patients received f2f CBT, which took on average 6:30 hours (SD 4:10 hours) per patient.

### Subgroup Analyses

#### Outcome of Face-to-Face Cognitive Behavioral Therapy After Internet-Based Cognitive Behavioral Therapy

In total, 95 patients received f2f CBT after I-CBT. As shown in Table 5, CIS fatigue was on average 5.6 points lower after f2f CBT, in comparison to after I-CBT, which was a significant change. Improvement on the SIP8 and SF-36 physical functioning was also statistically significant, whereas the SCL-90 score showed no statistically significant further improvement.

**Table 4:** Therapist time in stepped care.

Selected group	Stepped care–protocol-driven feedback						Stepped care–feedback-on-demand					
	n	Mean total time (hours)	Mean I-CBT^a^ time (hours)	Percentage of total time	Mean f2f CBT^b^ time (hours)	Percentage of total time	n	Mean total time (hours)	Mean I-CBT time (hours)	Percentage of total time (hours)	Mean f2f CBT time (hours)	Percentage of total time (hours)
Total group	121	9:10	4:04	44	2:06	23	121	8:30	2:29	29	2:54	34
No f2f CBT	81	7:00	4:06	59	0:00	0	67	5:11	2:19	45	0:00	0
Received f2f CBT	40	13:32	3:59	29	6:18	47	54	12:37	2:43	21	6:30	52

^a^I-CBT: internet-based cognitive behavioral therapy.

^b^f2f CBT: face-to-face cognitive behavioral therapy.

**Table 5:** Treatment effect of cognitive behavioral therapy after internet-based cognitive behavioral therapy.

Outcome measure	At T1 (after I-CBT^a^), mean (SD)	At T2 (after CBT), mean (SD)	*t* test (*df*)	*P* value
CIS^b^ fatigue severity (N=95)	42.99 (9.35)	37.39 (12.06)	4.901 (94)	<.001
SIP8^c^ (N=91)	1151.86 (660.76)	851.18 (673.74)	4.569 (90)	<.001
SF-36^d^ physical functioning (N=91)	71.54 (21.89)	77.03 (21.36)	-2.866 (90)	.005
SCL-90^e^ (N=86)	146.56 (33.29)	144.52 (46.01)	0.517 (85)	.607

^a^CBT: cognitive behavioral therapy.

^b^CIS: Checklist Individual Strength.

^c^SIP8: Sickness Impact Profile.

^d^SF-36: Medical Outcomes Survey Short Form-36.

^e^SCL-90: Symptom Checklist-90.

#### Differences Between First 240 and Last 123 Randomized Patients

There was a significant difference between the 2 groups regarding reduction of fatigue severity. In the first 240 patients, the change score was on average 16.4 points on the CIS fatigue severity subscale (SD 13.9). In the last 123 patients, the change score was 11.8 points (SD 11.3), *t*=3.374, *P*<.001. The differences between cohort 1 and 2 in change score for limitations (700.5 and 636.1, respectively), physical functioning (−15.7 and −14.5, respectively), and psychological distress (18.3 and 16.7, respectively) were not significant (*P*=.38, *P*=.59, and *P*=.74, respectively).

## Discussion

### Principal Findings

This study showed that I-CBT embedded in stepped care for chronic fatigue syndrome is noninferior to f2f CBT (TAU) in reducing fatigue severity. Treatment outcome of stepped care did not differ from TAU with respect to the level of disability, physical functioning, and psychological distress. The proportions of patients with clinically significant improvement of fatigue severity were equal for stepped care as well as TAU. Interestingly, this was despite the fact that approximately 50% of the patients who met the step-up criteria for f2f CBT after I-CBT did not step up.

For patients who did step up, it was found that f2f CBT after I-CBT led to a significant further improvement in fatigue severity and impairment. This suggests that stepped care with I-CBT as a first step is a viable treatment model for CFS. It was more time-efficient than usual care, as approximately 25% less therapist time was needed to deliver it. This is an important finding from a cost-effectiveness perspective, since therapist time accounts for a large proportion of the treatment costs in mental health care. Previous studies found stepped care, not including I-CBT, to be effective for CFS [20,41]. The findings of this study extend this observation and provide more insights into how stepped care can be offered in clinical practice. We found only 1 RCT that also investigated I-CBT embedded in stepped care. In patients with panic and social anxiety disorder, stepped care containing psychoeducation, I-CBT, and f2f CBT was compared with f2f CBT. Our findings are in line with this study: stepped care was noninferior to f2f CBT and was less time-intensive [42].

How therapist feedback during I-CBT influenced stepped care was explored. As in our previous study [17], we found that during I-CBT, less therapist time was needed in the feedback-on-demand than in the protocol-driven feedback format of I-CBT. However, when patients stepped up to f2f CBT, therapists spent similar time in both conditions. Although SC-feedback-on-demand remained more time-efficient, the difference between both became smaller and failed to reach significance. This might be explained by the fact that relatively more patients in the SC-feedback-on-demand format received f2f CBT, which led to an increase in invested time. Furthermore, since we did not power for a direct comparison between both conditions, we may need more patients to draw firm conclusions on this.

We also explored if the proportion of patients willing to step up after I-CBT differed in both the feedback formats. It was found that of the patients who needed to step up after I-CBT, the proportion of patients that received f2f CBT differed in favor of the feedback-on-demand format. However, this difference was not significant. It is important to know what prevented patients from stepping up. One plausible explanation could be that patients became less motivated after unsuccessful I-CBT [43,44]. In our study, we found that approximately 60% of the patients who declined did this because they did not want further therapy, although CFS remained a problem (eg, preoccupied with other matters in life, did not want to travel, or have no faith in further recovery). Interestingly, approximately 20% were satisfied with the result of I-CBT, although they were still severely fatigued and/or disabled. It is important to know why they were satisfied despite having severe complaints. Exit interviews could be used to investigate these matters further.

Although this study shows that I-CBT fits well into a stepped care model, it is problematic that many patients do not step up when this is needed. Although this did not lead to a lower proportion of improved patients (than in TAU), and it is uncertain whether these patients would have otherwise started and completed TAU, it is possible that some of these patients would have profited more if they had received f2f CBT. To improve the integration of I-CBT in clinical care, there are some options. An aim could be to increase the number of patients that step up for f2f CBT. For example, by stopping earlier with I-CBT when it appears to be ineffective, demoralization can be prevented. However, what is a reliable indicator of the need to step up is not known. In a process study on f2f CBT for CFS, it was found that in a substantial proportion of patients, symptoms did not decrease until after 3 to 4 months [45]. The absence of a change in fatigue-perpetuating cognitions and behavior may, therefore, be a more suitable indicator early on in therapy. Further research could focus on the predicting value of these perpetuators on treatment effect in I-CBT.

It is also possible to further improve I-CBT, so that fewer patients need to step up. An option would be to improve I-CBT by developing a more flexible version, in which the intensity of therapist guidance can be further varied. The integration of videoconferencing in I-CBT makes it possible to combine the advantages of f2f sessions and I-CBT. Perhaps this also can be *on demand*, as our study indicated that patients are able to determine how much guidance they need. It is also an option to predefine specific moments in therapy, in which video consults may have an added value, because some interventions are more difficult to deliver via the internet. For example, supporting reformulation of dysfunctional beliefs is probably easier in direct interaction than by email.

### Strengths and Limitations

A significant limitation of our study was that in TAU, one-third of the patients did not start the therapy. This may have reduced the treatment results in this reference group. The within-group effect size for fatigue severity in the TAU condition in our study fell outside the 95% CIs of 2 other CFS studies that had lower proportions of nonstarters [18,46]. Another possible explanation for the somewhat lower effect size in the TAU condition could be that during the study, the treatment center was moved to another university medical center. This led to organizational problems, such as uncertainty for patients about when treatment would start, changes in therapists, and substantially increased travel times for patients. There are indications that organizational features of a mental health center can influence the treatment outcome of behavioral interventions [47]. In all 3 conditions, patients who started their treatment during the last part of our study had a significantly lower reduction of fatigue.

Furthermore, a possible limitation is that the waiting list policy had changed during the study. However, retaining an unnecessarily long waiting list duration would not have been ethical. It was found that the duration of the waiting list had not influenced the treatment effect on reduction of fatigue.

Another limitation is that we did not provide data on dropout. It was difficult to produce a dropout definition that allowed for a comparison between all treatment arms. For TAU, termination of treatment before a certain number of sessions is often used as a criterion for dropout [48]. Dropout during I-CBT is difficult to measure reliably. Opening modules or logging in does not necessarily represent receiving treatment. The assessment of the therapist may also be unreliable if a patient does not request feedback or does not respond to an email; this does not have to imply that the patient dropped out of treatment as they still can follow the treatment via the internet. Unless patients actively report discontinuation of treatment, it is difficult to be certain whether someone dropped out. Furthermore, for stepped care, the number of f2f CBT sessions needed after I-CBT differs between patients, as the exposure to the content of I-CBT varies. This makes it difficult to define the minimal number of sessions that a patient has to follow before he or she is considered to have completed the treatment. More specific information on dropout, for example, by interviewing patients after they have terminated the treatment, would further aid comparison of the treatment formats.

Recently, new diagnostic criteria were proposed for CFS, by the US Institute of Medicine [33]. To aid comparison with other studies using this definition, we explored the proportion of our patients meeting SEID criteria and repeated our primary analysis with this subgroup of patients. We found the same pattern of results, suggesting that findings can probably be generalized to a significant subgroup of patients meeting the SEID criteria. It should, however, be noted that we did not assess orthostatic intolerance, which could have led to an underestimation of the proportion of patients meeting SEID in our sample.

There were important strengths of the study: it is one of the few RCTs that compared stepped care with I-CBT as a first-step treatment with usual care [19]. Our comparator was a high-intensity treatment that has proven to be effective in several RCTs [46]. Often, the efficacy of I-CBT or stepped care is compared with a no-treatment or low-intensity control group, which seriously limits the conclusions than can be drawn from these studies [15,43,49]. Another strength is that almost all patients completed postassessment on the primary outcome measure. Finally, the study included a large sample of patients, who underwent an extensive diagnostic procedure and were treated in a specialized tertiary treatment center by experienced, well-trained, and supervised therapists.

### Conclusion

In conclusion, this RCT showed that stepped care with I-CBT as the first and f2f CBT as the second step for chronic fatigue syndrome is noninferior to TAU in the reduction of fatigue severity. A substantial part of the patients did not need to step up after stepped care, which made treatment for them less intensive. For patients who needed to step up, f2f CBT led to an additional treatment effect. Stepped care took less therapist time to deliver. However, a substantial proportion of patients did not step up after I-CBT with limited results. Treatment efficacy can probably be improved when relatively more patients step up and by further developing the I-CBT.

## Appendix

Multimedia Appendix 1 CONSORT-EHEALTH checklist (V 1.6.1).

## Abbreviations

ANCOVA: analysis of covariance
CBT: cognitive behavioral therapy
CDC: Centers for Disease Control and Prevention
CFS: chronic fatigue syndrome
CIS: Checklist Individual Strength
f2f: face-to-face
I-CBT: internet-based cognitive behavioral therapy
ME: myalgic encephalomyelitis
RCI: reliable change index
RCTs: randomized controlled trials
SC: stepped care
SCL-90: Symptom Checklist-90
SEID: systemic exertion intolerance disease
SF-36: Medical Outcomes Survey Short Form-36
SIP8: Sickness Impact Profile
TAU: treatment as usual
